# Surgical treatment of isolated sternal fracture: serial of 10 cases

**DOI:** 10.1186/1749-8090-8-S1-P42

**Published:** 2013-09-11

**Authors:** PL Tahalele, Puruhito, A Prasmono, H Kusbijanto, H Soebroto, YE Sembiring

**Affiliations:** 1 Department of Surgery Division of Cardiothoracic and Vascular Surgery School of Medicine Airlangga University - Dr. Soetomo General Hospital Surabaya, Indonesia

## Background

Sternal fracture is uncommon injury following blunt chest trauma. They are usually caused by direct blunt trauma to the sternum, or most frequently car accident, including steering wheel direct blunt trauma to the sternum. Sternal fracture should be regarded as a hallmark of severe multiple injuries until proven otherwise. The purpose of this study was to describe general features correlated with etiology, sex, age, associated with injury, and treatment of sternal fracture.

## Methods

From June 1995 to June 2012, data of all patients suffering from sternal fracture were collected retrospectively.

## Results

Within fifteen years, eight patients with sternal fracture were treated. All of them were male with range of age 17 to 48 years old. In nine patients the fractures were caused by traffic accident, and in one patient it was due to falling from height. All of these cases were associated with other significant thoracic and extra thoracic injuries. The clinical manifestations of these cases mostly include anterior chest pain, tenderness, ecchymosis, swelling, and a palpable deformity and motion of the fracture fragments during respiration. The initial treatment of all these cases focused on the associated injuries. Later, in 10 cases, open reduction and internal fixation of the sternal fractures with wire were performed. After 6 month follow up, all the patients are alive and shows no complication.

## Conclusion

Surgical treatment in sternal fracture in 8 cases at Dr.Soetomo Hospital resulted in success outcomes.

